# Acceptability of two mobile applications to support cross-sectoral, person-centred and empowering stroke rehabilitation – a process evaluation

**DOI:** 10.1080/07853890.2024.2302979

**Published:** 2024-03-11

**Authors:** Mille Nabsen Marwaa, Susanne Guidetti, Charlotte Ytterberg, Hanne Kaae Kristensen

**Affiliations:** a Department of Physiotherapy Education, University College Southern Denmark, Esbjerg, Denmark; b Department of Clinical Research, Center for Innovative Medical Technologies, University of Southern Denmark, Odense, Denmark; c Department of Neurobiology Care Sciences and Society, Karolinska Institutet, Huddinge, Sweden; d Women’s Health and Allied Health Professionals Theme, Karolinska University Hospital, Stockholm, Sweden; e Geriatric Research Unit, Department of Clinical Research, University Hospital Odense, University of Southern Denmark, Odense, Denmark

**Keywords:** Stroke rehabilitation, person-centered rehabilitation, cross-sectoral rehabilitation, mobile applications, mobile apps, empowerment, occupational therapy, physiotherapy

## Abstract

**Aim:**

To evaluate the acceptability of two co-designed mobile applications *Mit Sygehus* [a knowledge-based solution] and *Genoptræn.dk* [a self-training solution] to support a cross-sectoral, person-centred and empowering stroke rehabilitation.

**Setting:**

The applications were implemented and tested throughout two stroke rehabilitation trajectories in Southern Denmark, comprising two acute, two sub-acute and two municipal stroke rehabilitation settings.

**Methods, participants and analysis:**

A process evaluation focusing on acceptability was conducted. Individual and dyadic interviews were performed with ten stroke survivors (three women and seven men, aged 50–84) with moderate stroke and seven significant others (five women and two men, aged 50–78) post-rehabilitation. A constructivist Grounded Theory analysis was used to explore what, why, when, and how the apps worked or did not work throughout the stroke rehabilitation trajectory and if adaptions were needed.

**Results:**

Participants found that *Mit Sygehus* provided adequate and sufficient knowledge and was easy to use, however, acceptability of *Mit Sygehus* declined throughout the rehabilitation process. Also, knowledge on ‘return-to-work’ and ‘re-gaining driver’s license/permission to drive’ needed to be developed. The content in *Genoptræn.dk* was perceived as acceptable, through content being person-centred, motivating and meaningful. *Genoptræn.dk* furthermore, supported the transfer between rehabilitation settings, provided a sense of progress throughout the rehabilitation process, facilitated positive habits regarding self-training, and relieved the burden on significant others. *Genoptræn.dk* was perceived most acceptable in the sub-acute rehabilitation setting and declined when rehabilitation continued in the municipal setting.

**Conclusion:**

Stroke survivors and their significant others found *Mit Sygehus* and *Genoptræn.dk* acceptable to support cross-sectoral, person-centred and empowering stroke rehabilitation, however acceptability declined throughout the rehabilitation process. Further investigations are required to determine how cognitive rehabilitation can play a greater role in app-supported stroke rehabilitation and how the need for more long-term follow-up can be supported.

## KEY MESSAGES

- By using process evaluation, the user acceptability of newly developed and complex interventions can be evaluated.
- ICT and app-based solutions can support cross-sectoral person-centred and empowering stroke rehabilitation.
- Therapists play an important role in implementing and supporting stroke survivors using app-based solutions to support person-centred and empowering stroke rehabilitation.

## Background

Rehabilitation after stroke should be person-centred, that is, based on the stroke survivors’ and significant others’ needs, goals, preferences, and resources [1–4], which supports empowerment [5, 6]. In the rehabilitation process, empowerment may be seen as an enabling process in which health professionals collaborate with stroke survivors and significant others, where the outcome is a stroke survivor and significant other with greater ability to exercise control, manage his/her condition, and make informed decisions [1, 4, 6, 7]. To be empowered, three components must be achieved: **intrapersonal** *(*perceives control, mastery, competence, being motivated, and showing initiative), **interactional** (has adequate relevant skills and knowledge to navigate in a specific context), and **behavioral** (empowered behaviors and actions taken to master or influence specific outcomes) [8]. Significant others are important because stroke might also affect their physical and emotional health [6, 9–11]. Therefore, person-centred and empowering rehabilitation must be continuously co-constructed in a partnership consisting of shared power, responsibility, and ownership for the rehabilitation process and outcomes [1]. Person-centred and empowering stroke rehabilitation should start as early as possible; be coherent and coordinated with smooth transitions between different rehabilitation settings; include goal-setting, intervention/training, support for social participation, discharge support, and continuous evaluation [1, 2, 6]. However, a well-known challenge to the quality of person-centred stroke rehabilitation is lack of coherence in cross-sectoral coordination and communication [1, 2, 12] and lack of person-centred initiatives [1, 11, 13, 14], leading to dis-empowered stroke survivors and significant others [5, 8, 11–13]. New technologies co-designed with all relevant stakeholders and rehabilitation settings to support these challenges are in demand [15, 16].

### ICT in stroke rehabilitation

The integration of information and communication technologies (ICT) and mobile-based applications (apps) are seen as both local and national visions and strategies to achieve equality and quality of health care, complement usual rehabilitation, deliver person-centred rehabilitation, and enhance independence and participation in everyday life [15–17]. ICT and apps (e.g. phone calls, messages, reminders, remote monitoring or intervention, text, videos, and images) may support the timing and delivery of relevant evidence-based health information [18–20], improve communication [21], support coordination, continuity, and coherence in the rehabilitation process [21, 22], increase accessibility to rehabilitation, reduce travel time and costs, and support follow-up after discharge [23]. Furthermore, stroke survivors’ motivation, engagement, and empowerment to independently perform person-centred exercises [19, 20, 23–26], and becoming more active in managing their own health and participating in everyday life and society may be supported by ICT and apps [18, 19, 24, 26–29] as well as reducing significant others’ strains and burden [30].

However, in Denmark, there are no overarching ICT/app-based solutions supporting cross-sectoral rehabilitation, which accommodates the need for coherent person-centred and empowering initiatives for stroke survivors and significant others [31]. In addition, existing ICT and app-based solutions focus mainly on isolated parameters of stroke rehabilitation [31], and there has been a lack of end-user involvement in the development, which reduces the acceptability and implementation of the solutions [21, 32–34].

### Aim

This study aimed to evaluate the acceptability of two co-designed app-solutions, *Mit Sygehus* and *Genoptræn.dk* to support cross-sectoral, person-centred and empowering stroke rehabilitation.

## Design and methods

We drew on the framework of the Medical Research Council (MRC) [35], who recommend that a process evaluation follows the development and testing of complex interventions before continuing to a full-scale implementation and evaluation [35, 36]. Qualitative results are recommended being published to support other researchers performing similar interventions [37]. The Standards for Reporting Qualitative Research (SRQR) guidelines were applied in this study [38] (see Supplementary Material).

### The rehabilitation settings

Stroke rehabilitation is recommended to take place in interdisciplinary specialized stroke units [12, 39]. In Denmark, stroke survivors are initially admitted to an acute hospital stroke unit (typically for a few days) [40, 41]. When required, rehabilitation continues in a sub-acute hospital stroke rehabilitation unit and/or in municipalities, that is, home-based or rehabilitation centers. Stroke survivors with more complex cognitive, emotional, mental, and/or physical difficulties most often need individualized and intensive interdisciplinary rehabilitation delivered in all three settings [39]. Municipal rehabilitation must be initiated within seven days after discharge from acute or sub-acute rehabilitation and typically continuous for three months [39]. Stroke severity is often defined as mild, moderate, or severe, using the Scandinavian Stroke Scale (SSS) and/or National Institutes of Health Stroke Scale (NIHSS) [39]. This study will mainly focus on stroke survivors in need of cross-sectoral rehabilitation in all three rehabilitation settings, that is, moderate to severe stroke.

### The intervention: Mit Sygehus and Genoptræn.dk

*Mit Sygehus* [in English: My Hospital] and *Genoptræn.dk* [in English: Rehab.dk] which are aimed at supporting cross-sectoral rehabilitation and involving multiple therapists, may be perceived as a complex intervention [36]. *Mit Sygehus* and *Genoptræn.dk* have been implemented in other patient trajectories in Southern Denmark; but have not yet been developed and implemented to support coherent cross-sectoral, person-centred and empowering stroke rehabilitation. Therefore, we co-designed the content of the two apps together with stroke survivors, significant others, physiotherapists (PTs) and occupational therapists (OTs) from all rehabilitation settings; one physician, one speech therapist, one representative from a patient organization, and three app developers [42] to accommodate end-user needs and to promote the uptake and implementation of the solutions. The developed content is described briefly below.

#### Mit Sygehus

**Figure 1.**
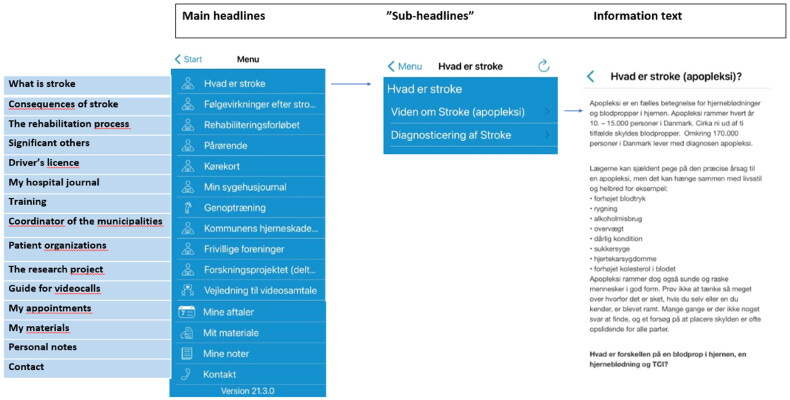
The content and interface of Mit Sygehus.

*Mit Sygehus* [in English, My hospital] is a free-of-charge app used in hospitals in southern Denmark to provide information related to the specific diagnosis. In an earlier co-design process [42] fourteen modules/headlines to support stroke rehabilitation were developed in *Mit Sygehus* each with ‘sub-headlines’ and information text: (1) what is stroke, (2) consequences of stroke, (3) the rehabilitation process, (4) significant others, (5) driver’s license, (6) my hospital journal, (7) training, (8) coordinator of the municipalities, (9) patient organizations, (10) the research project, (11) guide for videocalls, (12) my appointments, (13) personal notes and (14) contact (i.e. to the different rehabilitation settings) (see Figure 1).

#### Genoptræn.dk

**Figure 2.**
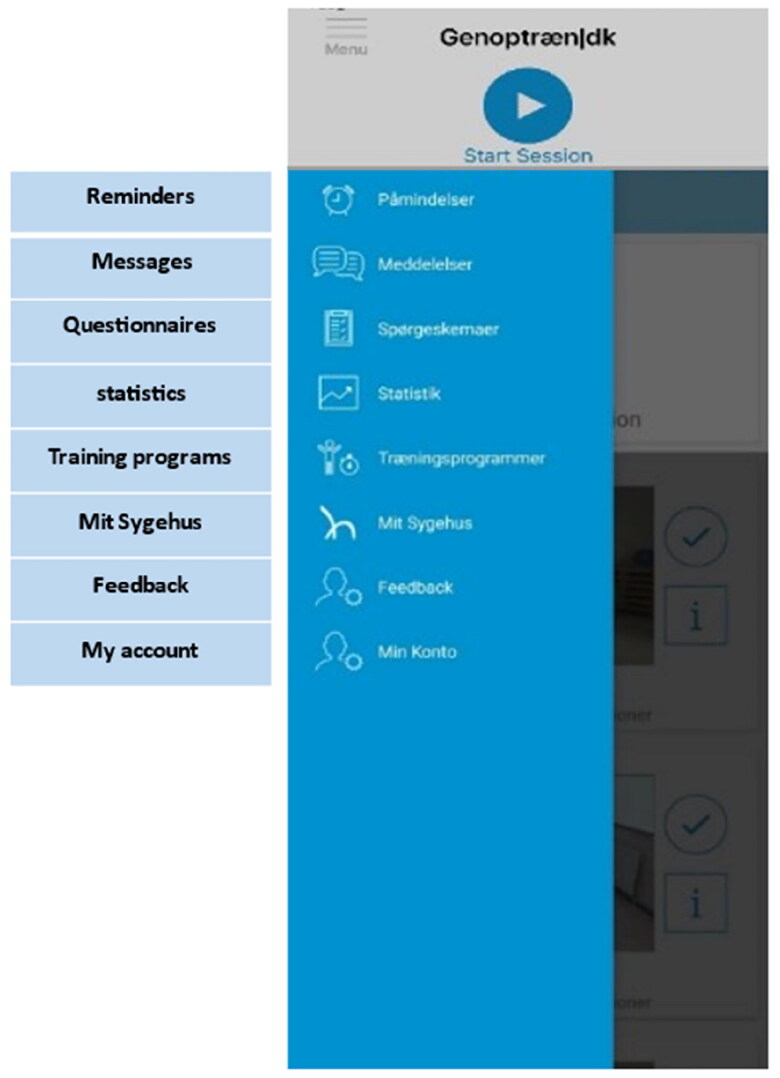
The content and interface of Genoptræn.dk.

*Genoptræn.dk* [in English, Rehab.dk] is currently being used in both hospital and municipal rehabilitation settings in Southern Denmark; however, the app has not previously been designed to support stroke rehabilitation. *Genoptræn.dk* is already integrated with *Mit Sygehus* meaning that the stroke survivor has all the content ‘in one place’ or can use the apps separately, depending on preference (see Figure 2). The app contains more than 700 generic video-recorded exercises that can be assigned by therapists to the personal app for stroke survivors. Content in the app is accessible to therapists in all rehabilitation settings, thus supporting cross-sectoral coherence, communication and documentation [43]. We additionally developed a ‘video recording’ function in *Genoptræn.dk* to accommodate earlier identified needs for more person-centred exercises and guidelines [11, 13, 32]. Further details of the co-design development process can be found elsewhere [42].

#### Implementation of Mit Sygehus and Genoptræn.dk and participants

The two app-solutions were implemented and tested through two stroke rehabilitation trajectories, comprising two acute stroke units (hospitals X and Y), two sub-acute stroke units (hospitals X1 and Y1), and two larger municipal stroke rehabilitation settings (municipalities X2 and Y2) in Southern Denmark. In each rehabilitation setting, one PT and one OT who also participated in the co-design process were engaged in the implementation. The PTs and OTs from the acute rehabilitation settings were responsible for identifying and including stroke survivors who were: (1) diagnosed with stroke; (2) living in one of the municipalities included in this study (municipality X2 or Y2); (3) in need of rehabilitation throughout all rehabilitation settings, that is, participants with more complex difficulties; (4) owning and using ICT such as a smartphone, tablet, and/or computer; and (5) preferably having a significant other to participate, that is, someone close to the stroke survivor offering support. Since acute stroke rehabilitation is often limited to a few days [40, 41], inclusion could also occur in sub-acute rehabilitation settings.

The PT or OT supported the stroke survivor and significant others to download and install both app solutions. Written instructions facilitated this. The therapists created access to the content in the apps by registering the stroke survivors’ social security numbers in the web-based back-end of the apps. If the stroke survivor consented, significant others could also receive access to the content of the apps on their own device.

Across the stroke rehabilitation settings, the therapists included apps to support the usual rehabilitation. *Mit Sygehus* was included if the stroke survivor and/or significant other expressed a need for supplementary information through the app. *Genoptræn.dk* was included when exercises, self-training, activities and/or guidelines were part of the intervention. Furthermore, if their performance status needed to be recorded, for example, gait function, ability to climb stairs, etc., and to support cross-sectoral communication. All included therapists had access to the stroke survivors’ content in *Genoptræn.dk*, which was adjusted throughout the rehabilitation process according to the stroke survivors’ goals, needs, and physical/cognitive functioning.

During the testing phase, the first author made participant observations during each stroke survivor’s rehabilitation process. The observations had three purposes: (1) to gain ongoing knowledge in each context and continue to maintain a relationship with the participating therapists; (2) to facilitate implementation of the two apps, for example, supporting the use of the apps and providing education when required; and (3) to get acquainted with the stroke survivor and significant others and obtain knowledge on their current use of the apps. Field notes on the therapists’ and stroke survivors’ use of the apps were made immediately after observation and were used to support post-intervention interviews.

The PTs and OTs from the acute setting delivered information to the first author on the participants’ stroke severity on admission (SSS or NIHSS). If multiple SSS scores were available, the first score was used (Table 1). In one case, the NIHSS score was provided and converted to SSS, as instructed by Gray et al. [44]. Therapists provided data on the length of stay in each rehabilitation setting and prints of the stroke survivors’ journals regarding the content and dates of the therapist interventions. Additionally, PTs and OTs informed the receiving stroke rehabilitation setting through secure crypted e-mails of their use or non-use of the two app-solutions.

**Table 1.** Participant characteristics.

	Patient, age (years)	Diagnoses and SSS	Significant other, age (years), relation	Duration of rehabilitation in each setting	Interview type and duration
1. Setting X	Beate, 65	Right side infarct SSS 46	Jonas, 72 Husband	Acute: 9 days Sub-acute: 7 days Municipal: 149 days (30 interventions)	Dyadic interview 97 min
2. Setting Y	Peter, 74	Left side infarct SSS 39*	Ella, 72 Wife	Acute: 10 days Sub-acute: 20 days Municipal: 91 days (3 interventions)	Dyadic interview 99 min
3. Setting X	Liam, 86	Right side infarct SSS: 43	Jane, 71 Wife	Acute: 2 days Sub-acute: 14 days Municipal: 123 days (2 interventions)	Dyadic interview 49 min
4: Setting Y	Jamie, 61	Left side cerebellum infarct SSS: 46	Betty, 56 Wife	Acute: 6 days Sub-acute: 14 days Municipal: 212 days (79 interventions)	Dyadic interview 47 min
5. Setting X	Robert, 58	Right side infarct SSS: 42	Ida, 61 Wife	Acute: 4 days Sub-acute: 15 days Municipal: 8 days (5 interventions)	Dyadic interview 83 min
6. Setting X	David, 50	Right side infarct SSS: 47	None Divorced	Acute: 7 days Sub-acute: 10 days Municipal: 28 July – **	Individual interview 49 min
7. Setting X	Erin, 80	Right side haemorrhage SSS: 29	Berry, 78 Husband	Acute: 6 days Sub-acute: 24 days Municipal: 29 days (unknown number of interventions)	Dyadic interview 17 min (telephone)
8. Setting X	Noah, 63	Right side infarct SSS: 31	None Divorced	Acute: 10 days Sub-acute: 57 days Municipal: continued in a private rehabilitation clinic	Individual interview 70 min (online)
9. Setting X	Edvin, 54	Right side infarct SSS: 54	Carol, 50 Wife	Acute: 8 days Sub-acute: 14 days Municipal: 211 days (39 interventions)	Dyadic interview 62 min
10. Setting Y	Anne, 84	Right side infarct SSS:49	None Widower	Acute: 3 days Sub-acute: 21 days Municipal: unknown duration (21 interventions)	Individual interview 44 min

*NIHSS score converted to SSS score [44].

**The stroke survivor had a second stroke just after being interviewed and just before being terminated from municipal rehabilitation, and thus continued his rehabilitation. Duration in municipal rehabilitation was therefore not obtained.

##### Sampling and included participants

A convenient sampling strategy was used [45, 46] with attention to variations in physical and cognitive deficits, as diversity is important when testing new solutions [37]. We planned to include 10 stroke survivors and 10 significant others, since 10 users would identify a minimum of 80% of the problems with the technology during testing and 20 users would identify 95% [37]. Ten stroke survivors and seven significant others were consecutively included from January 2022 to October 2022. Three stroke survivors, who were (1) not married, (2) divorced, or (3) widowers, did not wish to have a significant included. All invited stroke survivors and significant others participated in the study.

**Figure 3.**
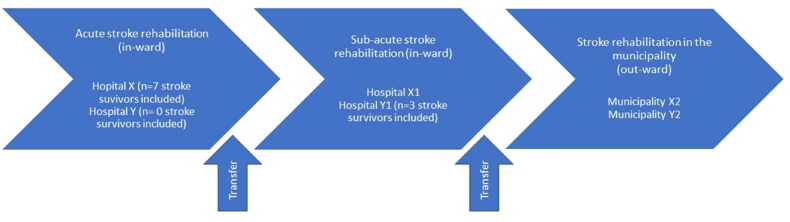
Included rehabilitation settings and included stroke survivors.

Figure 3 illustrates the cross-sectoral transfers when the stroke survivors needed rehabilitation in all three settings, that is, due to more complex declined functioning after stroke and the number of stroke survivors included from the acute and sub-acute rehabilitation settings. The participants’ characteristics are shown in Table 1.

### Process evaluation

Process evaluations are increasingly used to evaluate complex interventions and are less concerned about effectiveness outcomes but more concerned with understanding ***what***, ***why, how, for whom,*** and under ***what*** circumstances and in which contexts an intervention works, unexpected consequences, and whether adaptions are required [35, 36, 47, 48]. The evaluation of user ***acceptability*** is essential when conducting process evaluations of complex interventions [35, 49]. Acceptability, according to Sekhon et al. (2017), is a multi-faceted construct that reflects not only quantitative measures (if and how much the interventions were used), but also qualitative measures that capture the extent to which people delivering or receiving a healthcare intervention consider it appropriate to address their needs in a specific context [49], which impacts intervention implementation, uptake, adherence, intended outcomes, and overall effectiveness [50].

#### Logic model developed for this study

**Figure 4.**
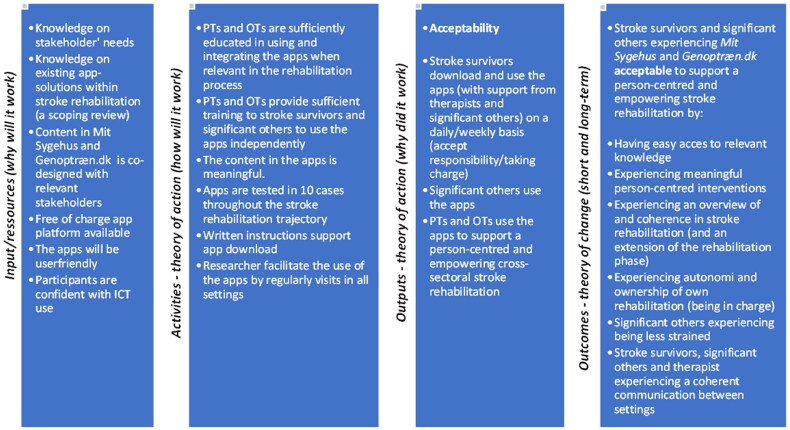
The final logic model.

A program theory (i.e. a theory of action and theory of change) was developed and visualized in a logic model to provide a better understanding of which mechanisms [8, 35, 36, 47, 51] that were expected to facilitate stroke survivors and significant others acceptability of *Mit Sygehus* and *Genoptræn.dk* to support cross-sectoral, person-centred and empowering stroke rehabilitation, which was the overall main outcome chosen for this study. Previous knowledge on stakeholders’ needs [11, 13, 32] and on existing app-solutions [31], having co-designed the content in the apps with stakeholders [42] were considered important mechanisms for achieving acceptability of the apps (see Figure 4).

### Ethical considerations

Written information in *Mit Sygehus* and oral information were delivered to stroke survivors and significant others by the PT and/or OT responsible for inclusion. Consent from all participants to participate were obtained in agreement with the Danish code of conduct and the Helsinki declaration. Ethical approval was obtained from the Danish Data Protection Agency (18/60280).

### Data generation

Based on the logic model, the following measures were chosen to evaluate the potential of *Mit Sygehus* and *Genoptræn.dk* to support cross-sectoral, person-centred and empowering stroke rehabilitation: 1) acceptability, that is, how the intervention was perceived in relation to practicality, barriers, facilitators of using the intervention, and how it supported the rehabilitation process, adaptions required and any unexpected consequences, and 2) what/which part of the apps were used. Table 2 presents the related research questions.

**Table 2.** Acceptability questions.

Research questions		
	Theory of action *- how* and *why* it worked/not worked	Theory of change *- outcomes*
Acceptability assessed qualitatively	Which inputs and actions were required for the apps to be used throughout the cross-sectoral rehabilitation processes? To what extent do the apps need to be refined or adapted to make them more acceptable, more relevant, or more useful to the specific contexts in which they are delivered? Were there any positive or negative unexpected consequences? Has implementation varied by settings/ contextual factors that needs to be considered?	What are the stroke survivors’ and significant others’ perspectives of the content in *Mit Sygehus* and *Genoptræn.dk*? What were stroke survivors’ and significant others’ experiences of *Mit Sygehus* and *Genoptræn.dk* to support a person-centred and empowering stroke rehabilitation*?*
Acceptability assessed quantitatively	Which parts of the apps were used by participants	

The concepts of person-centred rehabilitation [6] and empowerment [5, 8] supported the qualitative interviews, that is, elaborating questions on motivation, experienced competences, skills, knowledge, and behaviors in relation to the rehabilitation process were asked [5, 8]. Interviews were conducted by the first author in the stroke survivors’ home or in the rehabilitation setting of the municipality, in agreement with the participants’ preferences, when rehabilitation in the municipality had ended or was about to end. Data were gathered through dyadic interviews (stroke survivors and significant others). In three cases, individual interviews with the stroke survivors were performed, as they did not have a significant other included in the study. Open-ended questions were asked, for example, ‘tell me about your rehabilitation process’, ‘how and when was Mit Sygehus and Genoptræn.dk included in the rehabilitation process’, ‘what are your thoughts about the content in the apps?’ and ‘how did the apps support your rehabilitation process?’. Field notes from the observations made during the testing phase also supported the interviews. Interviews lasted 17–99 min.

Data on the quantitative use of the app were collected from the web-based back-end of the app, that is, which modules were used in the app and how many times.

Data from the OTs and PTs delivering the intervention, that is, acceptability, recruitment capability, implementation, adaptions needed and unexpected consequences [37], were gathered in focus group interviews after the testing period had ended and will be reported elsewhere.

### Data analysis

It is suggested that qualitative data used in process evaluations be analyzed continuously to allow learning to be successively implemented [36, 45], which was possible due to participants being consecutively included from January 2022 to October 2022. Thus, a constructivist Grounded Theory (GT) approach to analysis was appropriate [37, 45], as data generation and analysis should be carried out simultaneously. Conducting a constant comparison method, keeping memos, using theoretical sensitivity, and theoretical sampling are the steps used in GT [45, 46]. In a constructivist GT approach, data between the researcher and participant are constructed rather than theories being discovered [45].

**Figure 5.**
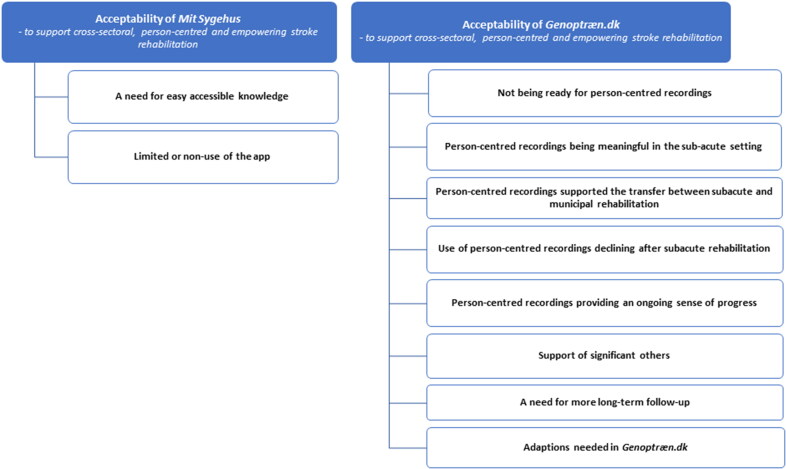
Results - acceptability of Mit Sygehus and Genoptræn.dk.

The interviews were transcribed verbatim by the first author. The next step of the analysis was open coding using the qualitative analysis tool NVivo [52]. Codes in this step were kept close to the data, sentence-by-sentence, or incident-by-incident and were made in Danish to make quick and spontaneous codes [53]. This was done by asking ‘what is going on here?’, ‘what does this mean?’ [45], and more processual questions like ‘who does what, when, where, how and with what consequences?’ [54]. The codes were then compared and organized into code groups (focused coding). A total of 20 code groups were constructed. After this stage, categories were developed by identifying the most significant content and the relationships between categories (theoretical coding). At this stage, the code groups were translated into English. Following this stage, the concepts of person-centred and empowering rehabilitation [1, 4–8] was included to support development of sub-themes describing the construct of ***acceptability*** of *Mit Sygehus* and *Genoptræn.dk* to support cross-sectoral, person-centred and empowering stroke rehabilitation, from the perspective of stroke survivors and significant others (Figure 5). Comparing, contrasting and (re)organizing the code groups, categories, themes and sub-themes was an iterative process. Throughout the data analysis process, the findings were discussed and reviewed by all authors.

## Results

Acceptability of the two apps *Mit Sygehus* and *Genoptræn.dk* to support person-centred and empowering stroke rehabilitation, from the perspectives of stroke survivors and significant others, is presented in two overall themes: ‘***acceptability*** of *Mit Sygehus*’ and ‘**acceptability** of *Genoptræn.dk*’ to support person-centred and empowering stroke rehabilitation. Mechanisms facilitating acceptability as well as contextual factors (i.e. rehabilitation phases) affecting acceptability, reasons for ‘non-use’ and suggestions for adaptions are elaborated on in respectively two and eight sub-themes (Figure 5).

### Acceptability of Mit Sygehus

*Mit Sygehus* was the most used app among the significant others, especially in the acute setting. Use of *Mit Sygehus* decreased when rehabilitation continued in the subacute and municipal setting. In the following sub-theme ‘a need for easy accessible knowledge’ mechanisms for acceptability of *Mit Sygehus* are elaborated on, as well as reasons for limited or non-use.

#### A need for easy accessible knowledge

Having all relevant knowledge/information in one place was valued by all participants using *Mit Sygehus*, although significant others seemed to use it more than the stroke survivors. ‘*I read it all, not just some of it, since it interested me… it is well explained in the app, and all is located in one place… it is ten times easier*’ (Jonas). The participants used *Mit Sygehus* to get informed, share knowledge, prepare for meetings, and re-read when needed. Participants explained mechanisms such as confidence in using ICT, the apps being user-friendly, containing relevant and evidence-based knowledge that was ‘just at hand’ and could be accessed when needed as crucial for acceptability. Most stroke survivors received support from therapists to install the two apps on their mobile phones. Four significant others (Jonas, Jane, Ella, Betina) had installed *Mit Sygehus* on their own mobile phone, only supported by written instructions.

#### Limited or non-use of the app

*Mit Sygehus* was mostly used in the acute (Carol, Jonas) and sub-acute (Jane) rehabilitation settings according to significant others. Carol explained that she gradually forgot about *Mit Sygehus* and instead used the Internet to gain the required knowledge. Statistical data from the back-end of *Mit Sygehus* showed that she had used the app on the day of installation and not again before the day of the interview. Significant others, furthermore, called for information on ‘return-to-work’ and how to ‘regain driver’s license’, especially during the later phases of rehabilitation.

Some stroke survivors explained they had only visited the *Mit Sygehus* a few times (Beate, Robert, Jamie, Noah) or not at all (Edvin, Erin, Liam, Peter, Anne). Reasons for limited or non-use were different, among others it had to do with the content being ‘confronting’ and reminding them of deficits they had/could have had. Lack of reading abilities, fine motor difficulties, and/or cognitive deficits, such as trouble remembering codes, were only mentioned in one case as barriers for using the app (Peter). Stroke survivors instead explained that they gained the needed knowledge through a significant other (Edvin, Beate, Peter, Jamie) or by receiving sufficient knowledge from health professionals (Liam, Edvin, Beate, Anne, Erin, Robert, Noah). However, statistical data from the back-end of *Mit Sygehus* showed that some stroke survivors who explained the non-use or limited use of *Mit Sygehus* during interviews had actually accessed the app a number of times throughout the rehabilitation process (Beate, Peter) or just a few times (Jamie, Robert, Noah) typically in the acute phase of rehabilitation (Table 3). Three stroke survivors (Liam, Erin, Edvin) had no longer *Mit Sygehus* installed on their mobile phone when the interviews were conducted, thus their ‘ID-number’ could not be identified, and eventual statistical data on the use of *Mit Sygehus* could not be identified from the back-end of the app (Table 3). Three significant others (Betty, Ida, and Berry) had either never heard about *Mit Sygehus* (Betty), failed to install the app (Ida) or did not wish to use apps (Berry). They explained gaining knowledge from the Internet, a booklet received during acute rehabilitation, through health professionals or through having previous knowledge about stroke. The ‘read-out-load’ function was not mentioned being used in any of the cases, most participant did not know it was an option, and/or did not perceive it as relevant.

**Table 3.** Stroke survivors’ and significant others’ use of Mit Sygehus and Genoptræn.dk.

Modules participants	Beate	Jonas husband	Peter	Ella wife	Liam	Jane wife	Jamie	Betty wife	Robert	Ida wife	David	Erin	Berry husband	Noah	Edvin	Carol wife	Anne
What is stroke	4				–	3	2	–		–	3	–	–		–	2	3
Consequences of stroke	26	14	8	2	–	7	1	–	1	–	3	–	–		–	7	
The rehabilitation process	22	13	10	2	–	5		–	3	–	7	–	–	2	–	1	
Significant others	10	23	1	13	–	4	1	–	5	–	10	–	–	2	–	1	1
Drivers licence	16	4	4	3	–	5	1	–	3	–	5	–	–	5	–	2	
My hospital jounal	48	4	4	2	–	2		–	6	–	12	–	–	3	–	2	2
Training	35	8	0	0	–	0	0	–	13	–	19	–	–	11	–	1	0
Genoptræn.dk app	50	–	10	–	8	–	15	–	69	–	2	17	–	15	1	–	15
Coordinator of the municipality	23	15		2	–	4	1	–		–	7	–	–	5	–	1	1
Patient organizations		5			–	2		–		–	1	–	–		–		
The research project	22	12	4		–	3	3	–		–	4	–	–		–		
Guide for videocalls	4			2	–	2		–		–		–	–		–		
My appointments	45	4		4	–	1	1	–	2	–	28	–	–	1	–	1	1
Personal notes	3	2	1	2	–	1	1	–	1	–	3	–	–		–		
Contact	4	2		1	–	1		–		–		–	–		–	1	

The numbers indicate number of times participants accessed the different modules.

‘- indicates that participants’ ID-number was in-retrievable, and therefore eventual use of *Mit Sygehus* could not be identified.

### Acceptability of Genoptræn.dk

*Genoptræn.dk* was the most used app among the stroke survivors, especially in the subacute setting. App installation mostly occurred in the acute setting of hospital X or hospital Y1, as hospital Y did not include any participants. Use of *Genoptræn.dk* decreased when rehabilitation continued in the municipal setting and was used even less when municipal rehabilitation had ended. In the following sub-themes acceptability of *Genoptræn.dk* in relation to the rehabilitation phases are elaborated on, as well as reasons for non-use and adaptions needed.

#### Not being ready for person-centred recordings

Mostly, stroke survivors did not perceive *Genoptræn.dk* as acceptable in the acute rehabilitation setting because of the short admission in this setting and the focus being more on acute care: ‘In the beginning you use a lot of energy on just being there, just sleeping and surviving…you need to be able to handle self-training before it makes sense’ (Noah). In only one case content in *Genoptræn.dk* was included in the acute setting (hospital X), where the stroke survivor had herself (Beate) initiated to do her self-training (facial exercises) and shared the videos with her husband, children, and grandchildren the same day they were provided in the app. This could have to do with this stroke survivor being the first participant in the study (and the therapist practicing using the app) and due to Beate experiencing a longer admission in the acute setting (nine days). Two other stroke survivors (David and Robert), perceived *Genoptræn.dk* could have been acceptable in the acute setting, to support daily training and waiting time before being transferred to a sub-acute setting, or before sub-acute therapists engaged in using the app to support the rehabilitation.

In order for *Genoptræn.dk* to be implemented in the acute setting, therapists are required to collaborate with the stroke survivor and the significant other regarding their wishes, needs and ‘readiness’ to use person-centred recordings to support early rehabilitation.

#### Person-centred recordings being meaningful in the sub-acute setting

Providing person-centred recordings in *Genoptræning.dk* to support self-training, based on individual and co-constructed goals, were overall meaningful and valued by the participants. The content in *Genoptræn.dk* were applied by the OT and or PT in the sub-acute setting and mostly contained video-recorded exercises and activities performed by stroke survivors themselves, such as gait, balance, strength (upper and lower limbs), fine motor, and facial exercises. In some cases, the gait performance was also recorded for status purposes: ‘Exercises were added in my app in the sub-acute setting…. It shows me in detail what I must do and how many times … it’s important that it is yourself on the recordings and not someone who has no problems’ (Robert). By using person-centred recordings, the stroke survivor was provided with a clear picture of ‘why, what and how’ to perform self-training which subsequently supported motivation: ‘It made it very clear for me which activities that were difficult, and thus needed to be trained. That motivated me’ (Edvin). In addition, the therapists in the sub-acute rehabilitation setting adjusted the content gradually to match individual goals and functioning: ‘They made the video recordings to support me … and when I didn’t need them anymore they were deleted or adjusted’ (Noah).

Participants explained that confidence in using ICT, the apps being user-friendly, and the content in *Genoptræn.dk* being relevant and meaningful as an important mechanism for acceptability: ‘It was easy to access and use… I just pushed there, and I am in’ (Beate). Most therapists instructed stroke survivors to perform daily self-training between other planned activities. However, some stroke survivors took initiative to perform the exercises also as compensation for cancelled training or if they felt restless and needed to do even more on their own: ‘I performed the exercises once a day, sometimes more, but the time was still long… sometimes I also went for a walk’ (Edvin).

Therapist-support, through education, facilitation and frequent integration of *Genoptræn.dk* during sub-acute rehabilitation setting also seemed to be important mechanisms, for stroke survivors’ motivation, active engagement and ownership of the content in the app. Frequent use also supported creating habits to perform daily self-training, and along the way it was not necessary to enter the app every time to perform self-training, since they could gradually be remembered and was made a habit: ‘I think it is easier to make a habit of it during a 3–4 week stay, than if he had been discharged after 4 days (Ida).

To additionally support self-training, therapists could use verbal instructions during person-centred recordings. Peter reported that audio instructions such as ‘slowly slowly’ from the PT during a ‘getting up and down from a chair’ exercise were stored and remembered long after rehabilitation had ended. Audio support also facilitated good quality exercises according to his wife Ella, especially if reading instructions were difficult.

#### Person-centred recordings supported the transfer between subacute and municipal rehabilitation

According to the participants, *Genoptræn.dk* supported the transfer from sub-acute to municipal setting, as well as the cross-sectoral communication between therapists and the ‘waiting time’ until municipal rehabilitation was initiated. One stroke survivor (Robert) experienced the therapist being prepared before the first municipal visit by beforehand watching the recordings made in sub-acute setting; and during the first visit, she made some adjustments according to his actual physical functioning. Robert imagined that watching the recording beforehand also supported the therapists: ‘I imagine they visit many patients during the day, and if they just watched the recordings beforehand, they would be prepared and maybe even save some reading time’ (Robert).

#### Use of person-centred recordings deteriorating after subacute rehabilitation

Stroke survivors and significant others explained that person-centred self-training were used less during municipal rehabilitation than in sub-acute rehabilitation: ‘I used to perform them a lot, now it is less frequent’ (Jamie). When discharged to the home setting, participants explained that everyday life activities took up more time, such as emptying the dishwasher, sweeping the terrace, cooking, fixing things in the garage, or gradually returning to work, and thus partially substituted using *Genoptræn.dk.* In addition, *Genoptræn.dk* appeared to be included less by therapists in municipal rehabilitation. Most stroke survivors did not remember having received any instructions on frequency regarding self-training during municipal rehabilitation, however, some stroke survivors found it supportive to receive a reminder, others performed exercises when it suited their everyday life or had made it a habit to perform them on specific weekdays: ‘I just performed them without reminders. I have made a habit out of doing my self-training on Tuesdays, Wednesdays, Fridays, Saturdays, and Sundays’ (David).

When performing person-centred self-training during municipal rehabilitation, stroke survivors explained that they did only access *Genoptræn.dk* occasionally to ensure that all exercises were remembered, and that the quality of the performance was in line with the recordings and guidelines given by the therapists: ‘Even after doing my exercises many times, doubt can still appear, and it is important for me to know if I perform the exercises correct. Then this solution is easy’ (Jamie). One stroke survivor explained that having self-training through *Genoptræn.dk* supported his engagement in everyday life, and that he and his wife could go camping or fishing when they wished to as the self-training was ‘along with him’ and could be performed everywhere (Robert). It was however also suggested that *Genoptræn.dk* was used more often during municipal rehabilitation and was continuously adjusted by the therapists to actual abilities to continuously support motivation and a sense of ongoing progress (Jamie and Robert).

Only two stroke survivors could imagine using the generic content in *Genoptræn.dk* (Noah and Jamie): ‘The better you get, maybe then it does not need to be yourself on the recordings’ (Jamie). Another stroke survivor declined municipal rehabilitation after the first visit because he felt that the oral information and guidelines received from hospital X1 were sufficient to manage on his own and without app support (Liam).

#### Person-centred recordings providing an ongoing sense of progress

The person-centred recordings seemed to provide an important ongoing ‘sense of progress’ for stroke survivors. And even though less frequent, many stroke survivors still occasionally performed their exercises also after municipal rehabilitation had ended, mostly to keep going, to ensure that the quality of the exercises was in line with recordings, and to perceive and watch progress made. Having ongoing access to the person-centred recordings in *Genoptræn.dk* and to ‘how it looked back then’ seemed to be important and continuously supported motivation, adherence and provided a sense of accomplishment for stroke survivors: ‘It gives you small updates, otherwise it could be hard to perceive progress, and you get small victories continuously, and not only at the end… also it is often my wife that thinks I have progressed; however, it is important that I myself get that experience… the recordings help me to experience that’ (Robert). Another stroke survivor explained: ‘I could see that it was myself on the recordings, and there was progress. Had there not been any progress I would have thrown the phone away’ (Edvin). Also, if motivation was low one day or for a period, watching the recording would remind them that things had progressed and that effort was worthwhile: ‘If you have an off day, you can access the app… it could be motivating’ (Beate). Also, it seemed that some stroke survivors were on their own able to adapt the use of *Genoptræn.dk* to match their current needs, for example, Robert had himself increased the number of repetitions on each exercise: ‘I still do them once in a while, but I have adjusted the number of repetitions, since I have progressed that much since they were recorded’.

#### Support of significant others

Most significant others found it rewarding to have the opportunity to watch person-centred recordings in *Genoptræn.dk*: ‘I watched the exercises on her mobile phone…otherwise, I would never have had the insight to what was relevant for her. It made me capable of supporting her better’ (Jonas). One significant other explained that she and their children felt more prepared when her husband came home for the weekends: ‘From these recording we prepared which tools were needed for him to perform his self-training at home…it was a kind of communication that helped me to prepare for the upcoming weekends’ (Carol).

Significant others only had access to *Genoptræn.dk* on their own ICT devices when using the stroke survivor’s login. This was only done by Ella, whose husband struggled with using ICT and apps. The others found it sufficient to have the opportunity to watch the content on the stroke survivors’ mobile phone, which together with provided reminders through the app also seemed to relieve the significant others of ‘being in charge’: ‘With the app, he just did it, no days of… it is his responsibility, I just ask about the app’ (Ida).

#### A need for more long-term follow-up

Even though participant expressed being well informed and receiving sufficient support from therapists during the rehabilitation process, several stroke survivors still called for follow-up support after municipal rehabilitation had ended. Some suggested a visit or just a phone call 3–6 months post-rehabilitation to support an ongoing sense of progress and status of performance: ‘I could imagine that a phone call after 6 months could get your thought back on how things actually are going… maybe some kind of re-test, or you do your self-training to get an idea of how things are actually going before all access is deleted in the app…that would be great’ (Robert). Some significant others confirmed a need for a more long-term follow-up and even though they knew that municipal rehabilitation would end after typically three months, they still experienced a sudden end of the rehabilitation process: ‘it would be great if the therapist returned or if we could go back… but it feels more like “three months has passed, over and out, you don’t have problems any longer”’ Jonas).

#### Adaptions needed in Genoptræn.dk

Two stroke survivors explained that they had not used *Genoptræn.dk* after discharge from sub-acute rehabilitation (Liam and Erin). Liam explained that he could remember the instructions received by the therapists, and thus he performed self-training without any other support. Erin explained that there were so many other things going on, and that fatigue and pain in her knee prevented her from performing self-training (waiting for knee replacement). She also explained that there were some challenges with *Genoptræn.dk* during the sub-acute rehabilitation, such as when performing the first exercise, it ‘jumped’ back to the first exercise instead of the next exercise in the app: ‘It annoyed me, and then I stopped using it’ (Erin). Two other stroke survivors (David and Jamie) also mentioned that obtaining an overview of how far they were in their self-training program was challenging. However, this was adjusted before their rehabilitation ended by supplying each exercise with a number, which solved the issue for them. However, some participants expressed a need for more flexibility in the exercises they wished to perform on that day. *Genoptræn.dk* supplies the exercises in the same order each time, and stroke survivors had to ‘tick-off’ each exercise before they could continue to the next or shift to another exercise program (if they had two or more programs, e.g. balance, lower limb strength, facial, upper motor exercises, etc.).

## Discussion

It is suggested that to make app-based solutions acceptable for supporting an empowering stroke rehabilitation, the developed solutions must be co-designed with relevant stakeholders, address the needs of stroke survivors and significant others [21, 55], and support a greater part of stroke rehabilitation and not only isolated parameters [31]. Furthermore, not only should the content of the app be evaluated but also the participants’ experiences with the developed solutions [56]. Therefore, this study aimed to evaluate the acceptability of two co-designed app-solutions, *Mit Sygehus* and *Genoptræn.dk* to support cross-sectoral, person-centred and empowering stroke rehabilitation by asking *what, why*, *how, for whom*, and under what circumstances and in which contexts the intervention works, as well as unexpected consequences and adaptions required [57].

Overall, the results showed that *Mit Sygehus* and *Genoptræn.dk* were acceptable and supported cross-sectoral, person-centred and empowering stroke rehabilitation; however, *Genoptræn.dk* was less acceptable in the acute setting, and the acceptability of *Mit Sygehus* declined throughout the rehabilitation process.

Important mechanisms for acceptability to support cross-sectoral, person-centred and empowering stroke rehabilitation were apps that were accessible, free-of-charge, user-friendly, participants being confident in ICT use, and content being relevant and meaningful. This is in line with previous studies showing that co-designed, evidence-based, and free-of-charge information and educational material delivered through an app [18] can support patient empowerment [19]. In this process evaluation, it appeared that the content in *Mit Sygehus* supported in gaining relevant knowledge throughout the rehabilitation process. It especially seemed to support significant others who, by reading and re-reading prepared for meetings with health professionals, supported their memory and were able to better support their stroke survivors. However, some significant others were not introduced to or insufficiently supported in using *Mit Sygehus.* If *Mit Sygehus* was only introduced and integrated in acute rehabilitation, it could seem to be a barrier for ongoing use throughout the rehabilitation process and thus a possible implementation failure. Also, a lack of knowledge on ‘return-to work’ and ‘re-gaining driver’s license/permission to drive car’ were mentioned as areas of improvement. The involvement of other sectors, such as the job sector, when trying to bridge ‘return-to-work’ is mentioned as challenging by the Danish whitepaper on rehabilitation [2]. Providing a designated ‘contact person’ that supports the coordination of post-stroke services and provides educational material is suggested to support the continuity of care [1, 58]. One initiative in Denmark, in trying to bridge this gap, is by employing ‘coordinators’ in municipalities [39]. Knowledge from the web-based *back-end* of *Mit Sygehus* identified that the module on ‘coordinator of the municipality’ had been used. However, this module primarily contained contact information to the coordinator and currently no detailed information regarding ‘return-to-work’.

Stroke survivors were less explicit about reading and using the content in *Mit Sygehus* even though statistical data from the ‘back-end’ of the app showed that some stroke survivors had accessed the content throughout the rehabilitation process. It seemed that stroke survivors’ need for information was lower than that of significant others, perhaps because significant others and therapists continuously supported them to obtain the needed information. Thus, the intrapersonal and interactional component of stroke survivor empowerment may have been supported adequately through significant other and therapist support. It is also likely that, at the time of the interview, they had forgotten when and for what they had used *Mit Sygehus*.

Stroke survivors seemed to gain more support from *Genoptræn.dk*. Stroke rehabilitation is complex, coordinated stroke rehabilitation is challenging and stroke survivors and their significant others have diverse individual needs regarding the rehabilitation process. [1, 2, 6]. Involving *Genoptræn.dk* in the stroke rehabilitation trajectory in this study seemed to support bridging the transfer between rehabilitation settings, especially between the sub-acute and municipal settings. Stroke survivors mostly did not feel ready to initiate self-training in the acute setting, although a few did, if the duration in the acute setting was prolonged more than the usual 2–3 days or if self-training was initiated late in the sub-acute setting. The inability to engage in and manage self-training in the very early stages after stroke has also been reported elsewhere [59, 60]. One explanation could be the short length of hospital stay, which challenges the identification of stroke survivors’ needs, goals and resources [59, 61]. However, this may negatively affect stroke survivors and significant others’ motivation, adherence [56] and empowerment [31]. Through person-centred rehabilitation, health professionals may be able to identify when stroke survivors are ready for engaging in co-constructed goal-setting and person-centred self-training to support stroke survivors’ empowerment in an acute setting.

Having person-centred exercises recorded in the sub-acute setting seemed to support motivation, ownership, and communication with significant others and therapists in the municipality. To our knowledge, there is limited use of person-centred video recordings to support self-training during stroke rehabilitation. Prescribed video recorded exercises are to our knowledge mostly generic recordings performed by healthy individuals without any deficits, which can affect stroke survivors’ motivation and make it hard to relate to as mentioned in this study. Although, a recent study protocol describe the use of people with stroke-related disabilities in their prescribed instructional generic video recordings to promote physical activity among stroke survivors [62], the results from our study indicate that recordings of the stroke survivor themselves support the experience of ‘clarity’, ‘necessity’, ‘ableness’, and a sense of progress.

The ongoing follow-up from therapists on the use of *Genoptræn.dk* during the rehabilitation process, including adjusting the content to match needs, goals, and stroke survivors’ abilities, seemed to be an important mechanism for their engagement, motivation, and adherence regarding person-centred self-training and seemed to support both intrapersonal, interactional and behavioural components of empowerment. Continuous co-constructed person-centred goal-setting and evaluation are an important part of rehabilitation [6, 64]; however, it is well known that stroke survivors struggle with setting goals and understanding their problems, especially in the acute setting [61], as mentioned above. A review on goal-setting found that stroke survivors tend to set broad long-term goals on an activity/participation level, whereas health professionals tend to set short-term and specific goals at the impairment level. Thus, conflicting priorities were mentioned as barriers to co-constructed person-centred goal-setting, in addition to limited participation from stroke survivors, and health professionals’ skills in goal-setting [59]. It seemed in this study that the person-centred recordings supported the co-constructed goal-setting process by making it clearer which activities and exercises were challenging and thus needed to improve both impairment and activity levels. Therefore, stroke survivors were supported in understanding their activity problems they needed to work on and how, which seemed to motivate them and supported the intrapersonal and interactional component of empowerment. Ongoing adjustment of the content in *Genoptræn.dk* to match current activity levels, new person-centred goals based on needs and preferences are known facilitators for co-construction and participation in the ongoing goal-setting process, which subsequently facilitates motivation and adherence to the chosen activities [59]. Additionally, experiencing a sense of forward momentum during stroke rehabilitation was important for stroke survivors’ motivation and engagement in this study, which was also found important in a review by Lloyd et al. from 2018 [63].

Therapists play an important role in teaching stroke survivors how to perform their self-training [24], and the connection with and encouragement from therapists has been shown to promote rehabilitation satisfaction [1], adherence [24], and confidence in their own abilities [61]. Although the duration of sub-acute rehabilitation was only 3–4 weeks, the daily and intensive use of *Genoptræn.dk* seemed to facilitate the development of positive habits regarding self-training, through support of intrapersonal and interactional component of empowerment, that continued in the municipality and post-rehabilitation where empowered behaviors were reported by the stroke survivors. In addition, the flexibility to perform self-training when fitted in daily life was valued by the participants in this study. Stabile habits can be created within a month through repeated active engagement in desirable behaviors such as self-training under consistent contextual circumstances [66], comprising tailored approaches aligned with specific and achievable person-centred goals [60]. Active engagement and participation in everyday life are often highly prioritized goals for stroke survivors [60]. Performing activities that are defined by the stroke survivor and not for the stroke survivor, which is not too easy or too difficult and can be achieved within a restricted time frame, positively affects the stroke survivors’ perceived competency in carrying out self-training [65].

Person-centred activities, exercises and guidelines also supported an overview of the progress made during the rehabilitation process in this study, when therapists across rehabilitation settings adjusted and deleted irrelevant content to continuously align with stroke survivors’ needs, wishes, and goals. This also meant that the content in *Genoptræn.dk* was diverse and matched individual stroke survivors’ abilities, which has been a challenge in another app-based study [56]. Continuous customized support to maintain motivation, self-management, and empowerment has been found to be important elsewhere [24, 56] when interventions are delivered through an app [31].

Although it is increasingly known that apps may support repetitive task-specific exercises and self-training in stroke rehabilitation [26], little evidence exists on apps supporting cognitive rehabilitation. In our study, *Genoptræn.dk* was mainly used to support physical function and facial deficits; however, a review by Zhou (2018) also found evidence for cognitive rehabilitation through apps, although this conclusion was based on only two studies [26]. Furthermore, a recent review concluded that ICT use could support cognitive function among older adults, such as memory, executive functioning and processing speed. Also, participants experienced that using ICT to support cognitive function drew less attention to their deficits, which made them feel less stigmatized [66].

Having therapists continuously follow-up seemed important for the participants, which is also supported elsewhere [29, 60]. However, stroke survivors in this study and in other studies [11, 13] also called for a follow-up three to six month post-rehabilitation to maintain a sense of progress and connectedness with therapists, which may also prevent a feeling of abandonment [1]. It is well known that rehabilitation needs may persist for at least one year after stroke [9] and that participation and engagement in everyday life is challenging to maintain when organized rehabilitation and support from health professionals ends [9, 11, 13, 14, 23], typically three months post-stroke [11, 13, 14]. Receiving reminders has been shown to support continued engagement in valued activities post-rehabilitation [67], and SMS reminders or phone calls until a year post-stroke may optimize satisfaction with the healthcare system, reduce significant others’ burden [30, 68], and decrease readmission rates [58]. In an ongoing study, weekly support through SMS for three months post-rehabilitation is currently being evaluated [68]. Thus, more long-term support for continued participation in meaningful activities after organized rehabilitation termination needs to be further investigated.

In this study, most participants who wished to use apps to support stroke rehabilitation found it easy to engage with *Mit Sygehus* and *Genoptræn.dk*. Although, patients with severe cognitive or physical disabilities have been reported to have trouble using apps independently [20, 58]. However, patient characteristics have not conclusively been able to explain differences in the perception of satisfaction with including technological solutions to support rehabilitation from the perspectives of health professionals [20] and stroke survivors [61]. Facilitators for using technologies to support rehabilitation are solutions that are simple and easy to use [20, 58], provide relevant information and insight into rehabilitation goals [20], can be customized to the patient’s needs, support self-training/home exercises and can be continued independently after discharge, [17, 20]. All these aspects have been complied with in this study and have been discussed above. In addition, contextual factors such as quality of coordinated communication and information [61], easy adoption in existing routines [1, 17], and sufficient technological knowledge and support for health professionals [21, 55] may facilitate satisfaction and uptake. These organizational factors will be discussed in a later manuscript describing the therapists’ acceptability.

In summary lessons learned from this process evaluation were that *Mit Sygehus* may be acceptable to support cross-sectoral, person-centred and empowering stroke rehabilitation, however content on ‘return-to-work’ and ‘re-gaining driver’s license/permission to drive’ needs to be developed and more stringent implementation is needed in the acute setting, that is continued in the other settings. *Genoptræn.dk* was perceived as acceptable to support cross-sectoral, person-centred and empowering stroke rehabilitation; through containing relevant, meaningful and motivating content and providing a sense of progress. However, integration already in an acute setting for participants expressing being ready for this could support person-centred and empowering stroke rehabilitation even more.

### Strengths and limitations of this study

A strength of this study was the developed logic model that described the mechanisms of actions needed for the acceptability of the app-solutions to support cross-sectoral, person-centred and empowering stroke rehabilitation. The logic model was continuously adjusted throughout the research process, which may have benefitted the participants. In addition, using dyadic interviews when possible was perceived as a strength, since the significant other and stroke survivors could collaborate on giving their insights, jogged each other’s memories, and together constructed suggestions for improvements. Furthermore, the fact that participants were interviewed around the time of post-rehabilitation was perceived as a strength regarding their willingness to be honest about *what* worked, *whe*n, and *how* it came to work. Insight into overall experienced empowerment would have been harder to understand if the timing of the interview was earlier. On the other hand, a later interview or a 3–6-month follow-up interview could potentially have elaborated on experienced empowerment in everyday life post-rehabilitation.

Making observations during the testing phase may be an important strength when evaluating new interventions to gain insight into what, how and why they worked, and what required further enhancements [36]. However, performing only one processual observational data collection for each included stroke survivor might also be perceived as a limitation because areas that may have needed further improvement may have been overlooked because they were forgotten at the time of the interview. However, PTs and OTs were also interviewed in focus groups, which provided insight into further adjustments needed before advancing to implementation and testing on a larger scale. In addition, more than one observational visit could have further facilitated the implementation of *Mit Sygehus* throughout the rehabilitation process by providing more support and education to therapists.

## Conclusion

Existing app solutions to support stroke rehabilitation mainly focus on isolated parameters of complex stroke rehabilitation, usually only assessment, or intervention/training. This study showed that *Mit Sygehus* and *Genoptræn.dk* may be acceptable from the perspectives of stroke survivors and significant others by: (1) focusing on a greater part of person-centred and empowering stroke rehabilitation; (2) providing easy and sufficient access to relevant and evidence-based information and education regarding stroke, deficits, and rehabilitation, and how to navigate within the healthcare system; (3) providing easy and meaningful person-centred intervention/training/activities/self-training based on stroke survivors’ and significant others’ actual and changing needs and goals; (4) supporting coherence between different rehabilitation sectors; and (5) continuous support from therapists. However, content on ‘return-to-work’ and ‘re-gaining driver’s license/permission to drive’ needs to be developed in *Mit Sygehus*. Also, more stringent implementation of *Genoptræn.dk* is needed in the acute setting as well as an investigation of how to provide cognitive rehabilitation in the app.

## Supplementary Material

Supplemental Material

## Data Availability

Can be shared upon request.

## Abbreviations

Apps: applications
ICT: information and communication technology
MRC: Medical Research Council
NIHSS: National Institutes of Health Stroke Scale
OT: occupational therapist
PT: physiotherapist
SSS: Scandinavian Stroke Scale

## References

[1] Jesus TS, Papadimitriou C, Bright FA, et al. Person-centered rehabilitation model: framing the concept and practice of person-Centered adult physical rehabilitation based on a scoping review and thematic analysis of the literature. Arch Phys Med Rehabil. 2022;103(1):1–18. doi:10.1016/j.apmr.2021.05.005. 34228955

[2] Maribo T, Ibsen C, Thuesen J, et al. Hvidbog om rehabilitering. Aarhus, Denmark: rehabiliteringsforum Danmark; 2022.

[3] Wade D. Rehabilitation – a new approach. Overview and part one: the problems. Clin Rehabil. 2015;29(11):1041–1050. doi:10.1177/0269215515601174. 26467940

[4] Bergström A, Koch L, Andersson M, et al. Participation in everyday life and life satisfaction in persons with stroke and their caregivers 3-6 months after onset. J Rehabil Med. 2015;47(6):508–515. doi:10.2340/16501977-1964. 25882897

[5] Barr PJ, Scholl I, Bravo P, et al. Assessment of patient Empowerment - A systematic review of measures. PLoS One. 2015;10(5):e0126553. Bond K, editor. doi:10.1371/journal.pone.0126553. 25970618 PMC4430483

[6] Wade DT. What is rehabilitation? An empirical investigation leading to an evidence-based description. Clin Rehabil. 2020;34(5):571–583. doi:10.1177/0269215520905112. 32037876 PMC7350200

[7] Cerezo PG, Juvé-Udina ME, Delgado-Hito P. Concepts and measures of patient empowerment: a comprehensive review. Rev Esc Enferm USP. 2016;50(4):667–674. doi:10.1590/S0080-623420160000500018. 27680054

[8] Zimmerman MA. Psychological empowerment: issues and illustrations. Am J Community Psychol. 1995;23(5):581–599. doi:10.1007/BF02506983. 8851341

[9] Ekstam L, Johansson U, Guidetti S, et al. The combined perceptions of people with stroke and their carers regarding rehabilitation needs 1 year after stroke: a mixed methods study. BMJ Open. 2015;5(2):e006784–e006784–e006784. doi:10.1136/bmjopen-2014-006784. PMC4330323 25678540

[10] Ytterberg C, von Koch L, Erikson A. Abandoned to the strains of daily life: a qualitative study of the long-term experiences in partners to persons after a mild to moderate stroke. Disabil Rehabil. 2017;41(6):649–655. doi:10.1080/09638288.2017.1401674. 29132222

[11] Marwaa MN, Ytterberg C, Guidetti S. Significant others’ perspectives on person-centred information and communication technology in stroke rehabilitation – a grounded theory study. Disabil Rehabil. 2019;42(15):2115–2122. doi:10.1080/09638288.2018.1555614. 30648452

[12] Wissel J, Olver J, Sunnerhagen KS. Navigating the poststroke continuum of care. J Stroke Cerebrovasc Dis. 2013;22(1):1–8. doi:10.1016/j.jstrokecerebrovasdis.2011.05.021. 21733720

[13] Gustavsson M, Ytterberg C, Nabsen Marwaa M, et al. Experiences of using information and communication technology within the first year after stroke – a grounded theory study. Disabil Rehabil. 2016;40(5):561–568. doi:10.1080/09638288.2016.1264012. 27976926

[14] Tistad M, von Koch L, Sjöstrand C, et al. What aspects of rehabilitation provision contribute to self-reported met needs for rehabilitation one year after stroke - amount, place, operator or timing? Health Expect. 2013;16(3):e24–35–e35. doi:10.1111/hex.12095. 23796012 PMC3883089

[15] Bech M, Bonde Klausen M, Buch MS. Tværsektoriel sundhedsforskning i danmark: aktører, fokusområder og forskningsbehov med udgangspunkt i region syddanmark. VIVE - Det Nationale Forsknings- og Analysecenter for Velfærd; 2022.

[16] Regioner D. EN KLAR RETNING FOR SUNDHEDSFORSKNING I DANMARK [Internet]. 2023. Available from: https://www.regioner.dk/media/23170/udspil-en-klar-retning-for-sundhedsforskning-i-danmark.pdf.

[17] Bezuidenhout L, Joseph C, Thurston C, et al. Telerehabilitation during the COVID-19 pandemic in Sweden: a survey of use and perceptions among physiotherapists treating people with neurological diseases or older adults. BMC Health Serv Res. 2022;22(1):555. doi:10.1186/s12913-022-07968-6. 35473602 PMC9038993

[18] Dubey D, Amritphale A, Sawhney A, et al. Smart phone applications as a source of information on stroke. J Stroke. 2014;16(2):86–90. doi:10.5853/jos.2014.16.2.86. 24949314 PMC4060267

[19] Kang YN, Shen HN, Lin CY, et al. Does a mobile app improve patients’ knowledge of stroke risk factors and health-related quality of life in patients with stroke? A randomized controlled trial. BMC Med Inform Decis Mak. 2019;19(1):282. doi:10.1186/s12911-019-1000-z. 31864348 PMC6925878

[20] Brouns B, van Bodegom-Vos L, de Kloet AJ, et al. Differences in factors influencing the use of eRehabilitation after stroke; a cross-sectional comparison between Brazilian and dutch healthcare professionals. BMC Health Serv Res. 2020;20(1):607. doi:10.1186/s12913-020-05457-2. 32611341 PMC7329422

[21] Nyman A, Zingmark M, Lilja M, et al. Information and communication technology in home-based rehabilitation – a discussion of possibilities and challenges. Scand J Occup Ther. 2023;30(1):14–20. doi:10.1080/11038128.2022.2046152. 35245989

[22] Brouns B, Meesters JJL, Wentink MM, et al. Why the uptake of eRehabilitation programs in stroke care is so difficult—a focus group study in The Netherlands. Implementation Sci. 2018;13(1):133. doi:10.1186/s13012-018-0827-5. PMC6206819 30373611

[23] Saywell N, Taylor D. Focus group insights assist trial design for stroke telerehabilitation: a qualitative study. Physiother Theory Pract. 2015;31(3):160–165. doi:10.3109/09593985.2014.982234. 25404160

[24] Simpson DB, Bird ML, English C, et al. Connecting patients and therapists remotely using technology is feasible and facilitates exercise adherence after stroke. Top Stroke Rehabil. 2020;27(2):93–102. doi:10.1080/10749357.2019.1690779. 31762412

[25] Silveira TM, Tamplin J, Dorsch S, et al. Let’s improvise! iPad-based music therapy with functional electrical stimulation for upper limb stroke rehabilitation. Australian Journal of Music Therapy. [Internet]. 2018;29:1–16. Available from: https://www.austmta.org.au/journal/article/let%E2%80%99s-improvise-ipad-based-music-therapyfunctional- electrical-stimulation-upper

[26] Zhou X, Du M, Zhou L. Use of mobile applications in post-stroke rehabilitation: a systematic review. Top Stroke Rehabil. 2018;325(7):1–11. doi:10.1080/10749357.2018.1482446. 30209991

[27] Fell N, True HH, Allen B, et al. Functional measurement post-stroke via mobile application and body-worn sensor technology. Mhealth. 2019;5:47–47. doi:10.21037/mhealth.2019.08.11. 31728382 PMC6851460

[28] McKay FH, Cheng C, Wright A, et al. Evaluating mobile phone applications for health behaviour change: a systematic review. J Telemed Telecare. 2018;24(1):22–30. doi:10.1177/1357633X16673538. 27760883

[29] Paul L, Wyke S, Brewster S, et al. Increasing physical activity in stroke survivors using STARFISH, an interactive mobile phone application: a pilot study. Top Stroke Rehabil. 2016;23(3):170–177. doi:10.1080/10749357.2015.1122266. 27077973

[30] Bakas T, Clark PC, Kelly-Hayes M, et al. Evidence for stroke family caregiver and dyad interventions: a statement for healthcare professionals From the American heart association and American stroke association. Stroke. 2014;45(9):2836–2852. doi:10.1161/STR.0000000000000033. 25034718

[31] Marwaa MN, Guidetti S, Ytterberg C, et al. The use of mobile and web-based applications to support rehabilitation after stroke: a scoping review. JRM [Internet]. 2022 [cited 2022 Apr 4]. Available from: https://medicaljournalssweden.se/jrm/article/view/452. 10.2340/jrm.v54.452 PMC10348057 35174871

[32] Marwaa MN, Kristensen HK, Guidetti S, et al. Physiotherapists’ and occupational therapists’ perspectives on information and communication technology in stroke rehabilitation. Disability and Rehabilitation. 2020. 10.1371/journal.pone.0236831 PMC7454973 32857781

[33] Bate P, Robert G. Experience-based design: from redesigning the system around the patient to co-designing services with the patient. Qual Saf Health Care. 2006; 115(5):307–310. doi:10.1136/qshc.2005.016527. 17074863 PMC2565809

[34] Ramey L, Osborne C, Kasitinon D, et al. Mobile health technology in rehabilitation. Phys Med Rehabil Clin N Am. 2019;30(2):485–497. doi:10.1016/j.pmr.2018.12.001. 30954161

[35] Skivington K, Matthews L, Simpson SA, et al. Framework for the development and evaluation of complex interventions: gap analysis, workshop and consultation-informed update. Health Technol Assess. 2021;25(57):1–132. Sep doi:10.3310/hta25570. PMC7614019 34590577

[36] Moore GF, Audrey S, Barker M, et al. Process evaluation of complex interventions: medical research council guidance. BMJ. 2015;350(6):h1258–h1258. doi:10.1136/bmj.h1258. 25791983 PMC4366184

[37] O’Cathain A, Hoddinott P, Lewin S, et al. Maximising the impact of qualitative research in feasibility studies for randomised controlled trials: guidance for researchers. Pilot Feasibility Stud. 2015;1(1):32. doi:10.1186/s40814-015-0026-y. 27965810 PMC5154038

[38] O’Brien BC, Harris IB, Beckman TJ, et al. Standards for reporting qualitative research: a synthesis of recommendations. Acad Med. 2014;89(9):1245–1251. doi:10.1097/ACM.0000000000000388. 24979285

[39] Danish Health Authority. Anbefalinger for tværsektorielle forløb for voksne med erhvervet hjerneskade - apopleksi og transitorisk cerebral iskæmi (TCI) - traume, infektion, tumor, subarachnoidalblødning og encephalopati [Internet]. 2020. Available from: https://www.sst.dk/-/media/Udgivelser/2020/Hjerneskade/Anbefalinger-forloeb-hjerneskade.ashx?la=da&hash=A7A96AC766D6AA68D26F32B96C0015BF828C93FF.

[40] Tistad M, Ytterberg C, Sjöstrand C, et al. Shorter length of stay in the stroke unit: comparison Between the 1990s and 2000s. Top Stroke Rehabil. 2012;19(2):172–181. doi:10.1310/tsr1902-172. 22436365

[41] Stroke sengeafsnit. Aarhus Universitetshospital Internet]. 2023. Available from: https://www.auh.dk/afdelinger/neurologi/stroke-sengeafsnit/.

[42] Marwaa MN, Guidetti S, Ytterberg C, et al. Using Experience-based Co-design to develop mobile/tablet applications to support a person-centred and empowering stroke rehabilitation [Internet]. In Review; 2023 [cited 2023 Jul 5]. Available from: https://www.researchsquare.com/article/rs-3075410/v1. 10.1186/s40900-023-00472-z PMC10463694 37620982

[43] genoptræn.dk. Genoptræn|dk – din digitale træningsmakker [Internet]. 2019. Available from: https://www.syddansksundhedsinnovation.dk/projekter/genoptraen-dk-din-digitale-traeningsmakker/.

[44] Gray LJ, Ali M, Lyden PD, et al. Interconversion of the national institutes of health stroke scale and scandinavian stroke scale in acute stroke. J Stroke Cerebrovasc Dis. 2009;18(6):466–468. doi:10.1016/j.jstrokecerebrovasdis.2009.02.003. 19900650

[45] Charmaz K. Constructing grounded theory. 2nd edition. London ; Thousand Oaks, Calif: sage; 2014. 388. p. Introducing qualitative methods).

[46] Qureshi HA, Ünlü Z. Beyond the paradigm conflicts: a Four-Step coding instrument for grounded theory. Int J Qual Methods. 2020;19:160940692092818. doi:10.1177/1609406920928188.

[47] Funnell SC, Rogers PJ. Purposeful program theory: effective use of theories of change and logic models. 1st ed. San Francisco, CA: jossey-Bass; 2011. 550. p.

[48] Schultz Petersen K, Maindal HT, Ledderer L, et al. Komplekse interventioner: udvikling, test, evaluering og implementering [in english: complex interventions: development, test, evaluation and implementation]. Aalborg Universitetsforlag; 2022.

[49] Sekhon M, Cartwright M, Francis JJ. Acceptability of healthcare interventions: an overview of reviews and development of a theoretical framework. BMC Health Serv Res. 2017;17(1):88. doi:10.1186/s12913-017-2031-8. 28126032 PMC5267473

[50] Sekhon M, Cartwright M, Francis JJ. Development of a theory-informed questionnaire to assess the acceptability of healthcare interventions. BMC Health Serv Res. 2022;22(1):279. doi:10.1186/s12913-022-07577-3. 35232455 PMC8887649

[51] Fumagalli LP, Radaelli G, Lettieri E, et al. Patient empowerment and its neighbours: clarifying the boundaries and their mutual relationships. Health Policy. 2015;119(3):384–394. doi:10.1016/j.healthpol.2014.10.017. 25467286

[52] Lumivero. NVIVO 13 [Internet]. 2018. Available from: www.lumivero.com.

[53] Charmaz K. Grounded theory in global perspective: reviews by international researchers. Qualitative Inquiry. 2014;20(9):1074–1084. doi:10.1177/1077800414545235.

[54] Timmermans S, Tavory I. Data analysis in qualitative research: theorizing with abductive analysis. Chicago: university of Chicago Press; 2022.

[55] Brouns B, Meesters J, Wentink M, et al. Factors associated with willingness to use eRehabilitation after stroke: a cross-sectional study among patients, informal caregivers and healthcare professionals. J Rehabil Med. 2019;51(9):665–674. doi:10.2340/16501977-2586. 31414140

[56] Epalte K, Tomsone S, Vētra A, et al. Patient experience using digital therapy “vigo” for stroke patient recovery: a qualitative descriptive study. Disability and Rehabilitation: assistive Technology. 2020;18(2):175–184. doi:10.1080/17483107.2020.1839794. 33155507

[57] Moore GF, Evans RE. What theory, for whom and in which context? Reflections on the application of theory in the development and evaluation of complex population health interventions. SSM Popul Health. 2017;3:132–135. doi:10.1016/j.ssmph.2016.12.005. 29302610 PMC5742639

[58] Deutschbein J, Grittner U, Schneider A, et al. Community care coordination for stroke survivors: results of a complex intervention study. BMC Health Serv Res. 2020;20(1):1143. doi:10.1186/s12913-020-05993-x. 33341112 PMC7749985

[59] Plant SE, Tyson SF, Kirk S, et al. What are the barriers and facilitators to goal-setting during rehabilitation for stroke and other acquired brain injuries? A systematic review and meta-synthesis. Clin Rehabil. 2016;30(9):921–930. doi:10.1177/0269215516655856. 27496701 PMC4978164

[60] Warner G, Packer T, Villeneuve M, et al. A systematic review of the effectiveness of stroke self-management programs for improving function and participation outcomes: self-management programs for stroke survivors. Disabil Rehabil. 2015;37(23):2141–2163. doi:10.3109/09638288.2014.996674. 25579669

[61] Lindblom S, Flink M, Sjöstrand C, et al. Perceived quality of care transitions between hospital and the home in people with stroke. J Am Med Dir Assoc. 2020;21(12):1885–1892. doi:10.1016/j.jamda.2020.06.042. 32739283

[62] Thurston C, Bezuidenhout L, Humphries S, et al. Mobile health to promote physical activity in people post stroke or transient ischemic attack – study protocol for a feasibility randomised controlled trial. BMC Neurol. 2023;23(1):124. doi:10.1186/s12883-023-03163-0. 36978045 PMC10043533

[63] Lloyd A, Bannigan K, Sugavanam T, et al. Experiences of stroke survivors, their families and unpaid carers in goal setting within stroke rehabilitation: a systematic review of qualitative evidence. JBI Database System Rev Implement Rep. 2018;16(6):1418–1453. doi:10.11124/JBISRIR-2017-003499. 29894410

[64] Beshears J, Lee HN, Milkman KL, et al. Creating exercise habits using incentives: the trade-off Between flexibility and routinization. Manage Sci. 2021;67(7):3985–4642. doi:10.1287/mnsc.2020.3706. 35001975 PMC8734590

[65] Maes S, Karoly P. Self-Regulation assessment and intervention in physical health and illness: a review. Applied Psychology. 2005;54(2):267–299. doi:10.1111/j.1464-0597.2005.00210.x.

[66] Wilson SA, Byrne P, Rodgers SE, et al. A systematic review of smartphone and tablet use by older adults With and Without cognitive impairment. Innov Aging. 2022;6(2):igac002. Pak R, editor. doi:10.1093/geroni/igac002. 35243008 PMC8889997

[67] Lindqvist E, Borell L. Computer-based assistive technology and changes in daily living after stroke. Disabil Rehabil Assist Technol. 2012;7(5):364–371. doi:10.3109/17483107.2011.638036. 22149354

[68] Fysioterapeuter D. Træning skal skræddersyes [in english: training must be individualized]. The Physiotherapist. 2022;8:17–22.

